# A comparative study between traditional and sports school uniforms on cardiorespiratory and muscular fitness and waist-height-to-ratio in adolescents

**DOI:** 10.3389/fpubh.2023.1213403

**Published:** 2023-06-28

**Authors:** Carlos Cristi-Montero, Ricardo Martínez-Flores, Felipe Porras, Kabir P. Sadarangani, Gerson Ferrari, Nicolas Aguilar-Farias, Inacio Crochemore M. Silva, Tomas Reyes-Amigo, Fernando Rodriguez-Rodriguez

**Affiliations:** ^1^IRyS Group, Physical Education School, Pontificia Universidad Católica de Valparaíso, Valparaíso, Chile; ^2^Escuela de Kinesiología, Facultad de Salud y Odontología, Universidad Diego Portales, Santiago, Chile; ^3^Universidad Autónoma de Chile, Santiago, Chile; ^4^Escuela de Ciencias de la Actividad Física, el Deporte y la Salud, Universidad de Santiago de Chile (USACH), Santiago, Chile; ^5^Department of Physical Education, Sports, and Recreation, Universidad de La Frontera, Temuco, Chile; ^6^Escola Superiorde Educação Física, Programa de Pós Graduação emEducação Física, Universidade Federal de Pelotas, Pelotas, Rio Grande do Sul, Brazil; ^7^Observatório de Ciencias de la Actividad Física, Departamemto de Ciencias de la Actividad Física, Universidad de Playa Ancha, Valparaiso, Chile

**Keywords:** education, public health, students, policy, mental health, physical activity

## Abstract

**Background:**

Improving health of children and adolescents is crucial for their overall development. Therefore, it is essential to explore factors that may influence their health at both the public health and school system levels.

**Objective:**

This study compares physical fitness components and waist-height-to-ratio (WHtR) in adolescents according to school uniforms, namely the traditional uniform (i.e., shirt and school necktie in boys and skirt and blouse in girls) and the sports uniform (i.e., polo shirts or t-shirts and sport or short trousers). Additionally, it seeks to investigate potential differences in these measures based on sex and school type (i.e., public, subsidized, and private).

**Methods:**

This cross-sectional study used data from the Chilean national learning outcome assessment system (SIMCE)–2014 and involved 8,030 adolescents. Cardiorespiratory fitness (CRF) and muscular fitness (MF) were measured. WHtR was assessed as a health cardiovascular indicator. Mixed models and ANCOVA were performed to compare uniform types, adjusting for multiple covariates. value of p and effect size (ES) was used to establish significant results.

**Results:**

Overall, sports uniforms (SU) were linked to higher CRF (*p* < 0.001) than the traditional uniform (TU). Boys from private schools wearing SU presented higher CRF (*p* = 0.016; ES = 0.37), and a positive trend was observed for MF (*p* = 0.645; ES = 0.21). In subsidized, a trend was found in CRF (*p* = 0.005; ES = 0.16). Girls wearing SU from private schools showed a positive trend in CRF (*p* = 0.167; ES = 0.28). Trends in WHtR were found in both sexes from private (*p* = 0.555; ES = 0.24; *p* = 0.444; ES = 0.25, respectively).

**Conclusion:**

Wearing SU seems a promissory alternative to promote healthy physical fitness and body composition at the educational level. However, the relationship between higher physical activity and CRF, MF, and lower WHtR due to SU must be verified. Finally, when deciding to implement this measure, special attention must be paid to boys from public schools and girls from all types of schools.

## Introduction

It has been established for decades that traditional school uniforms (i.e., shirt and school necktie in boys and skirt and blouse in girls) play a relevant role in enhancing discipline, academic performance, and removing social differences (1, 2). The utilization of traditional uniforms holds a longstanding tradition in various countries, including England, the United States (in private schools), New Zealand, and Singapore (3–6). Indeed, some of these governments declare to use them to improve student behavior, discipline, and academic performance (2, 7). However, recent evidence indicates that there are no differences between traditional (TU) and sports uniforms (SU) (i.e., polo shirts or t-shirts and sport or short trousers) in academic and cognitive performance, perception of bullying, and school discrimination in adolescents (1, 8).

In this sense, in recent years, different investigations have been carried out on the influence of school uniforms on the general well-being of students, with special attention to physical activity and health (1, 9–12). Accumulating evidence suggests a notable association between the type of school uniform and student performance in fitness-related activities (1). In particular, studies consistently indicate that TU is associated with poorer outcomes in terms of fitness levels, physical activity, and overall health (1, 6, 13). In contrast, SU positively correlates with improved physical fitness (especially cardiorespiratory fitness), increased physical activity participation, and improved general health indicators (8–10). The mechanism underlying the association between SU and improved fitness can be attributed to several factors. Firstly, the freedom of movement afforded by this type of uniform enables students to engage in physical activities more comfortably and effectively (8, 9, 11). The absence of restrictive clothing, such as skirts or ties, allows for a broader range of motion, facilitating participation in exercise and active play (10, 12). Secondly, adopting SU may even improve students’ active commuting time (13). These results underscore the importance of considering the design and functionality of school uniforms as crucial factors in promoting students’ physical well-being (1).

Globally, it is possible to observe an unfavorable trend in children and adolescent’s levels of obesity, physical inactivity, and fitness (14–16). Moreover, these three modifiable risk factors have been related to adverse health and educational outcomes. For instance, children living with obesity and those who do not meet physical activity and physical fitness recommendations present a higher prevalence of cardiometabolic diseases and lower cognitive and academic performance (17–19). Therefore, low-cost and feasible strategies at public and education levels are demanded worldwide to revert this complex landscape and reduce health inequalities (20). In this way, modifying school uniforms could be one feasible alternative (8) and simultaneously would support students’ perceptions that they would be more active if they wore sports uniforms (9).

In the case of Chile, children and adolescents show one of the highest prevalence worldwide of overweight and obesity, physical inactivity, and low physical fitness level (21). Besides, most Chilean adolescents wear TU at schools, but some have gradually decided to switch to SU. A plausible reason is that, due to the lockdown by COVID-19, most schools have performed classes virtually; thus, it was not necessary to wear TU, so principals and families have begun to question its use mainly for economic concerns (8). It should be noted that in many schools where TU is required is also indispensable to buy various types of uniforms, which represents a significant economic burden for families (22). Moreover, no clear educational advantages are observed in schoolchildren using TU compared to SU (8).

In the Latin-American context, countries present some of the highest inequality rates related to income distribution worldwide (23), showing relevant social gaps such as gender and access to physical activity and sports (20, 24). In Chile, family socioeconomic status is highly predictive of the school type their children attend; thus, low-, middle- and high-socioeconomic status families send their children to public, subsidized, and private schools, respectively (25). Therefore, it is possible that uniforms could affect boys’ and girls’ physical fitness and fatness differently according to school type. Nonetheless, this hypothesis has not been explored in the literature.

This issue is relevant to analysis because studies have shown that wearing TU can inhibit self-expression (22) and limit schoolchildren’s physical activity, especially of girls (26), which can increase gender gaps in early stages (10) and increase health inequality (20, 27). Thereby, this study aimed to compare cardiorespiratory and muscular fitness and waist-height-to-ratio in adolescents according to school uniforms (TU vs. SU). Additionally, we explore differences by sex and school type (i.e., public, subsidized, and private).

## Methods

### Study design

This cross-sectional study used data from the Chilean national learning outcome assessment system (SIMCE) – 2014 in Physical Education. The SIMCE is organized by the Chilean Ministry of Education and aims to measure aspects of physical fitness, with several tests validated nationally and internationally (28). The SIMCE was carried out between November 17 and December 5, 2014. The database is available on request from the Chilean Ministry of Education. The present study was performed according to STROBE guidelines.

### Study population

The general inclusion criteria were girls and boys from 8th grade having a valid physical fitness evaluation. Overall, the Academic SIMCE involved 30,187 adolescents from 15 regions of Chile, including 370 establishments (private, subsidized, and public) (28). The exclusion criteria were (a) schools located in Juan Fernández, Rapa Nui, and Antarctica, (b) schools with less than 10 students in 8th grade, (c) schools with more than four classes per level. Additionally, establishments that had had accidents in previous applications were also excluded (to protect the students’ safety) and those that had been evaluated the previous year (since establishments are not repeated to avoid a possible overload of evaluations) (29). Nonetheless, only a subsample participated in the Physical Education SIMCE. The sample size was defined as 374 schools, gathering a total of 15,696 8th grade students, distributed equally in each of the 15 regions of the country, and with approximately equal representation by gender (48% female and 52% male) (29). Of them, 8,030 met the inclusion criteria. Our statistical criteria were to keep adolescents with the most frequent physical test (i.e., the standing long jump) and after imputing remaining tests. More information about this procedure in the statistical analysis item.

Table 1 shows the descriptive participant characteristics, of them a total of 64.1% wore a TU (21.1, 38.1, and 5.0% were from the public, subsidized, and private schools), while 35.9% wore SU (13.8, 18.7, and 3.3% were from the public, subsidized, and private schools).

**Table 1:** Descriptive participant characteristics.

Variable	All (*n* = 8,030)	Sports uniforms (*n* = 2,882)	Traditional uniforms (*n* = 5,148)	Value of *p*
Sex (*n*, %) Boys	4,220 (52.6%)	1,482 (18.5%)	2,738 (34.1%)	0.129
Girls	3,810 (47.4%)	1,400 (17.4%)	2,410 (30.0%)	
Age (years)	13.8 ± 0.7	13.8 ± 0.7	13.8 ± 0.7	0.942
Weight (kg)	58.4 ± 11.7	58.1 ± 11.5	58.5 ± 11.8	0.162
Height (cm)	160.9 ± 8.4	160.8 ± 8.1	161.0 ± 8.5	0.307
Peak height velocity (y)	0.82 ± 0.8	0.84 ± 0.8	0.82 ± 0.8	0.359
Cardiorespiratory fitness (stage)	4.8 ± 2.3	4.9 ± 2.4	4.8 ± 2.3	**0.045**
Standing long jump (cm)	149.5 ± 31.9	149.6 ± 32.9	149.5 ± 31.3	0.934
Waist-to-height ratio	0.45 ± 0.05	0.45 ± 0.05	0.45 ± 0.05	**0.014**

Data are presented as mean ± SD or frequency (%) according to variable features. In bold, significant differences between SU and TU groups. Significant values were established at *p* < 0.05.

### Type of school uniforms

Our team telephonically contacted school coordinators or principals to know the student’s uniform type worn in 2014. The TU was defined as a polo shirt or shirt (with school tie), sweater or blazer, and pants, and for girls, a skirt and blouse, and sweater or blazer, both with school shoes (usually black leather) (8). TU is worn every day of the week except for Physical Education classes, where adolescents wear SU. At the same time, SU was defined as sports clothing such as polo shirts or t-shirts and sports pants or shorts (jeans were also included in this category due to their widespread use and sports shoes) (8). Schools stated that their students wear sports uniforms every day.

### Measurements

All tests were applied from Monday to Friday in the morning in a single session where anthropometric assessments were evaluated first and after physical fitness tests. The evaluators primarily consisted of Physical Education teachers selected by the institution that organized the national evaluation. All of them were trained previously by specialized personnel from the Ministry of Education of the Government of Chile (28).

### Anthropometric assessments

Weight was measured with a digital scale where adolescents had to climb barefoot on the scale and remain for 5 sec without moving, ideally with shorts and a light shirt. Value in kilograms was recorded. While height was measured with a portable stadiometer where adolescents had to climb barefoot and stand with their back to the height rod, the Frankfurt plane was aligned (30). Waist circumference was evaluated with a tape measure at the narrowest point between the lower costal arch and the iliac crest (30). Finally, the waist-to-height ratio (WHtR) was obtained by dividing the waist circumference by the subject’s height (30).

### Cardiorespiratory fitness

The cardiorespiratory fitness (CRF) was evaluated with the 20-m shuttle run test (28, 31) and carried out at the end of the evaluation session (criterion validity, *r* = 0.89) (31, 32). It is used to assess the maximum aerobic power, that is, the capacity of the body to supply the necessary oxygen to the muscles during physical exertion. In this test, the participant must move along a lane between two parallel lines located 20 meters apart to the rhythm of a sound pulse that accelerates progressively. This test was selected because it measures cardiorespiratory fitness in large populations and also a large body of evidence supports it (31, 33). The initial velocity was 8.5 km/h and increased by 0.5 km/h every minute (28, 31). The test ended voluntarily when the adolescent was fatigued or unable to reach the line twice. The stage number reached was recorded.

### Muscular fitness

Muscular fitness (MF) was evaluated with the standing long jump test. It is used to assess the strength of the lower body (criterion validity, *r* = 0.80) (28, 31, 34). The standing long jump test is traditionally used to assess lower body strength and power (31). This test has been used by different international batteries to establish muscular fitness (e.g., ALPHA-fitness test battery, PREFIT battery) (31, 35). The adolescent was positioned behind a starting line, feet apart, and jumped as far as possible on the oral signal, landing with both feet simultaneously. Measurement was performed twice (with at least one-minute rest between attempts). The greatest distance was recorded in centimeters (28, 31).

### Covariates

Age, sex, maturation, school vulnerability index (SVI), hours of Physical Education classes, features of Physical Education classes (boys and girls together or separately), and location of the establishment (urban or rural) were included in the analyzes due to their relevance and relationship to the outcomes. It has been stated that age, sex, and maturation are relevant factors associated with the amount of body fat and as strong predictors of obesity in childhood (27). The maturation was calculated according to the peak height velocity (PHV), subtracting the PHV age from the chronological age (36). In addition, the SVI was included as a proxy for a socioeconomic factor at the school level. This Chilean index measures the socioeconomic vulnerability of students who attend schools with partial or total state financing (subsidized and public schools, respectively) based on the educational level of the parents-guardians, the state of health, physical well-being, and students’ emotional and school location. In addition, two features of the Physical Education classes were considered, the first was the total hours of classes during the week, a relevant indicator related to physical fitness and adiposity (37), and the second was whether boys and girls performed classes together or separately. Finally, the location of the educational establishment (rural or urban) was considered since previous literature indicates that it is associated with the risk of obesity (38).

### Statistical analysis

Previously to imputing, outliers were detected and were replaced according to the Tukey method through the “funModeling” R package (39). This method distinguishes probable outliers to be treated, which lie outside the outer fence. Thus, the distribution’s inner fence is defined as 1.5 x inter-quartile range below Q1 and 1.5 x inter-quartile range above Q3. Each outlier value was explored and studied to be removed (i.e., measurement error) or replaced by the interquartile value. Afterward, missing data were imputed based on the non-parametric missing value method using random forest through the “missForest” R package (40). Missing data ranges were between 1.8% (body weight) to 19.2% (CRF), and the estimation error was 0.06% for numeric variables and 0.00% for factors. The central limit theorem for sample sizes over 500 participants was considered (41) and Q-Q plots (quantile-quantile plot) were used to check normality visually.

Mixed model analyzes were performed to establish differences between uniform types in CRF, MF, and WHtR. To compare the likelihood of a model with the effect included vs. a model with the effect excluded, the likelihood-ratio test (LTR) for the random effect was estimated. A significant value indicates that the model with random effect is significantly better (in terms of likelihood) than the model without the cluster, and the interclass correlation coefficient (ICC) was estimated. School type (k = 3; public, subsidized, and private) was used as a cluster (random effect). Post-hoc tests were estimated using the Holm correction for multiple comparisons. ANCOVAs were used to explore differences by sex and school type. Significant values were established at *p* < 0.05, while a statistical trend was declared when one indicator was significant and the other one was not (e.g., *p* > 0.05 and Cohen’s d ≥ 0.2). Cohen’s d was interpreted as no effect (<0.2), small (0.2 < 0.5), medium (0.5 < 0.8), and large (≥0.8) (42). Furthermore, the collinearity of the model was corroborated (VIF between 1.02 and 2.25). All models were adjusted for multiple covariates mentioned above and were conducted using mixed models with the statistical software Jamovi version 2.3.18.

## Results

**Figure 1:**
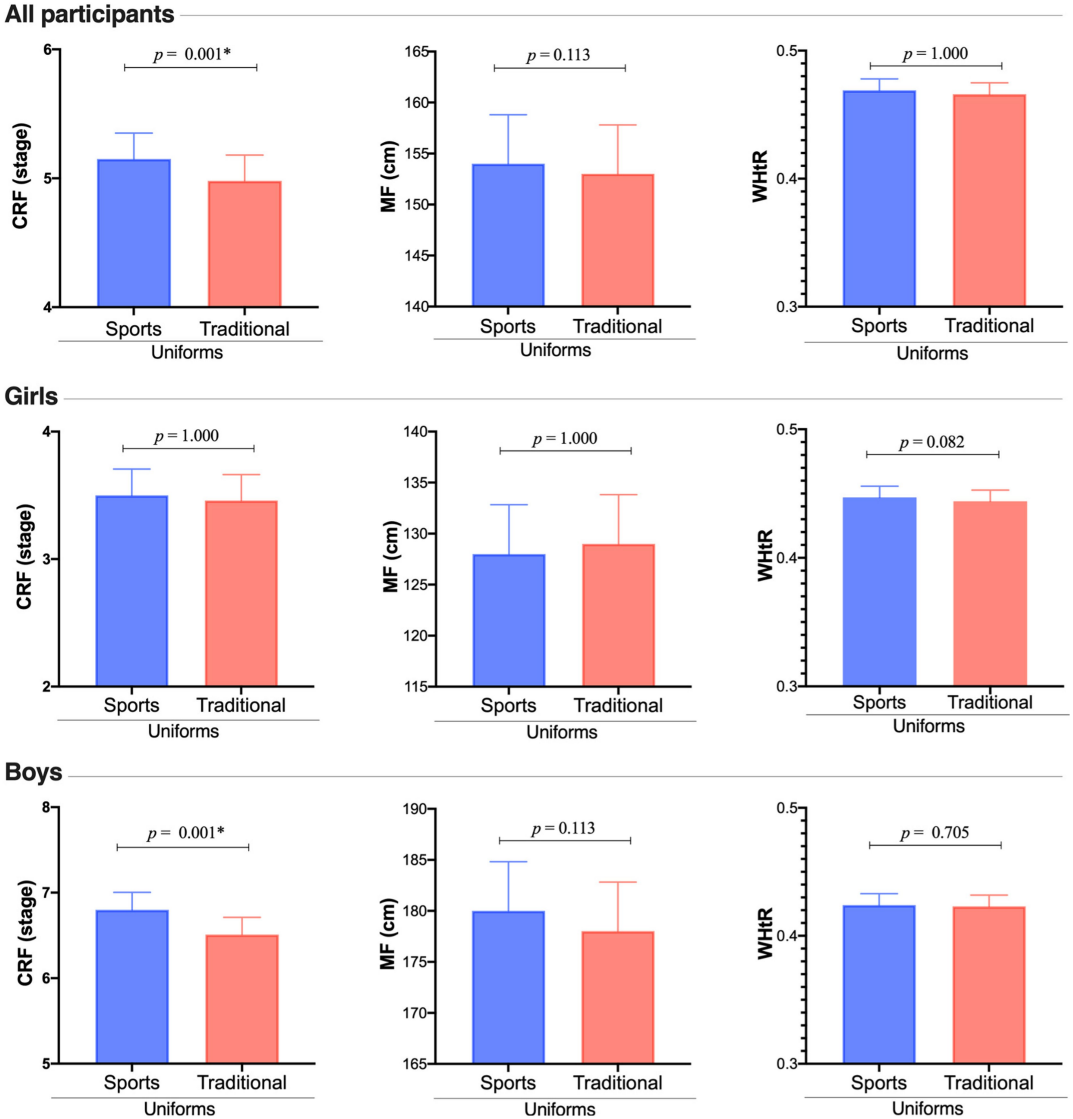
Differences in CRF, MF, and WHtR according to uniform types and separated by sex (estimated marginal means and standard error). *Significant difference between the two types of uniforms.

Figure 1 shows significant differences between uniform types in all participants’ CRF (LTR = 46.2; *p* < 0.001; ICC = 0.030). Nonetheless, this significant difference is observed in boys (*p* = <0.001; ES = 0.37) but not in girls (*p* = 1.000; ES = 0.28). Additionally, no differences were found for WHtR in all participants (LTR = 122; *p* < 0.001; ICC = 0.064), neither in boys nor girls (ES = 0.24–0,25 respectably). Similar results were detected for MF (LTR = 176; *p* < 0.001; ICC = 0.098).

Differences in CRF, MF, and WHtR according to uniform types and separated by sex (estimated marginal means and standard error). *Significant difference between the two types of uniforms.

Tables 2–4 show the ANCOVAs results, analyzing differences by sex and type of schools in CFR, MF, and WHtR, respectively. Overall, significant differences were obtained in boys wearing SU compared to TU from private schools (*p* = 0.016; ES = 0.37). In particular, five trends were found on CFR, MF, and WHtR; CFR in boys from subsidized schools (*p* = 0.005; ES = 0.016) and girls from private schools (*p* = 0.167; ES = 0.28), MF only in boys from private schools (*p* = 0.645; ES = 0.21), and WHtR in boys (*p* = 0.555; ES = 0.24) and girls (*p* = 0.444; ES = 0.25) from private schools.

**Table 2:** Differences in CFR according to sex and school uniform type.

					95% Confidence interval			
Sex	Uniform type	School type	Mean	SE	Lower	Upper	*P*-values	ES
Boys	Traditional	Public	6.38	0.078	6.23	6.53	1.000	0.07
	Sport	Public	6.53	0.092	6.35	6.71		
	Traditional	Subsidized	6.33	0.720	6.19	6.47	**0.005**	0.16
	Sport	Subsidized	6.64	0.086	6.47	6.81		
	Traditional	Private	6.65	0.148	6.36	6.94	**0.016**	**0.37**
	Sport	Private	7.36	0.168	7.03	7.69		
Girls	Traditional	Public	3.20	0.084	3.04	3.37	1.000	0.00
	Sport	Public	3.21	0.094	3.03	3.40		
	Traditional	Subsidized	3.35	0.075	3.20	3.49	1.000	0.01
	Sport	Subsidized	3.33	0.089	3.15	3.50		
	Traditional	Private	3.75	0.144	3.47	4.04	0.167	**0.28**
	Sport	Private	4.30	0.184	3.94	4.66		

The value of *p* for Holm correction. ES: effect size (Cohen’s d). In bold significant or trend differences according to both value of *p* and Cohen’s d.

**Table 3:** Differences in MF according to sex and school uniform type.

					95% Confidence interval			
Sex	Uniform type	School type	Mean	SE	Lower	Upper	*P*-values	ES
Boys	Traditional	Public	174	1.022	172	176	1.000	0.05
	Sport	Public	175	1.215	172	177		
	Traditional	Subsidized	174	0.943	172	176	0.770	0.07
	Sport	Subsidized	176	1.136	174	178		
	Traditional	Private	180	1.947	177	184	0.645	**0.21**
	Sport	Private	186	2.202	181	190		
Girls	Traditional	Public	122	1.102	120	125	1.000	0.02
	Sport	Public	122	1.241	119	124		
	Traditional	Subsidized	124	0.983	122	126	1.000	0.00
	Sport	Subsidized	124	1.170	122	127		
	Traditional	Private	143	1.896	139	147	1.000	0.09
	Sport	Private	145	2.415	140	150		

The *p*-value for Holm correction. ES: effect size (Cohen’s d). In bold significant or trend differences according to both *p*-value and Cohen’s d.

**Table 4:** Differences in WHtR according to sex and school uniform type.

					95% Confidence interval			
Sex	Uniform type	School type	Mean	SE	Lower	Upper	*P-*values	ES
Boys	Traditional	Public	0.428	0.002	0.423	0.432	0.588	0.11
	Sport	Public	0.434	0.002	0.429	0.440		
	Traditional	Subsidized	0.432	0.002	0.428	0.437	1.000	0.09
	Sport	Subsidized	0.433	0.002	0.428	0.438		
	Traditional	Private	0.421	0.004	0.412	0.429	0.555	**0.24**
	Sport	Private	0.407	0.005	0.397	0.417		
Girls	Traditional	Public	0.472	0.002	0.467	0.447	1.000	0.09
	Sport	Public	0.477	0.003	0.471	0.483		
	Traditional	Subsidized	0.447	0.002	0.473	0.482	1.000	0.00
	Sport	Subsidized	0.482	0.002	0.477	0.487		
	Traditional	Private	0.477	0.004	0.438	0.455	0.444	**0.25**
	Sport	Private	0.432	0.005	0.421	0.443		

The *p*-value for Holm correction. ES: effect size (Cohen’s d). In bold significant or trend differences according to both *p*-value and Cohen’s d.

## Discussion

We aimed to establish differences between two physical fitness components and a central fatness indicator according to adolescents’ school uniform types. Our findings showed that wearing SU was linked to higher CRF, particularly in boys from private schools, although also a trend was displayed in girls and boys from private and subsidized schools, respectively. In MF positive trend was observed in boys from private. Finally, trends in WHtR (lower values) were found in boys and girls from private schools. To the best of our knowledge, this study seems to be the first to describe a favorable association between SU wearing with CRF, MF, and WHtR in a large adolescent sample. Due to this study’s novelty findings, it was impossible to compare with others in the literature. However, it is possible to theorize that CRF was higher in schools where adolescents wear SU because they play and move more.

In this sense, some studies support this conjecture showing that children wearing SU reduce sedentary time, play more, and increase incidental physical activity (1, 8–10). Otherwise, children declare that TU is a barrier to playing more during lunchtime (12) and affects their physical activity at school (8). Thus, reducing schoolchildren’s physical activity would reduce CRF (43). It is relevant to highlight that higher CRF in children and adolescents has been associated with better academic achievement, cognitive performance, and cardiovascular health (17–19); hence, implementing SU as the primary school uniform could help to promote and meet global education and health indicator (44). To demonstrate the practical significance of this finding on CRF, we can consider the following exercise. If we estimate the maximal oxygen consumption using Leger’s equation, we observe a difference of 2.7 mL of oxygen per minute per kilogram of weight between the SU and TU, favoring SU. Although this difference may appear visually slight, it should not be underestimated, as it corresponds to 74.3% of 1 metabolic equivalent (MET). It is worth noting that previous evidence suggests that a mere increase of 1 MET can significantly impact children’s cardiovascular health, weight management, and blood pressure reduction (45, 46).

Regarding sex differences, two epidemiological studies showed that boys accumulate more physical activity than girls (24). Consequently, wearing SU could be considered as a facilitator factor related to higher physical activity and CRF in boys. However, the evidence seems to be not conclusive in girls. Previous studies found that girls were significantly more active at recess, lunch, and overall than boys when wearing SU (9). Nonetheless, it has also been reported that the change of uniform in girls did not improve their physical activity (10), supporting -to some extent- our main finding on CRF and MF. We hypothesize that this may be due to gender differences and gaps in physical activity, therefore, more studies (i.e., observational and interventions) are needed to determine how modifying school uniforms in girls could affect their physical activity and fitness, and also consider other aspects such as self-motivation and self-image perception (24, 26).

According to school type (i.e., a proxy of Chilean socioeconomic status), our results show that the benefits of wearing SU on CRF are observed only in boys from subsidized and private schools and in addition, two positive trends in girls (CFR and WHtR) only in private schools (i.e., middle- to high-socioeconomic status). Extensive research has consistently highlighted a significant relationship between socioeconomic status and various cardiometabolic risk factors, including physical inactivity, poor fitness levels, and obesity (47). Individuals from lower socioeconomic backgrounds face a greater likelihood of experiencing adverse cardiometabolic outcomes compared to those with higher socioeconomic status (48, 49). This association can be attributed to a multitude of intertwined factors, lower socioeconomic individuals often encounter economic barriers that limit their access to resources and opportunities for engaging in regular physical activity, thereby leading to sedentary lifestyles (47, 50, 51). Children from families with low-socioeconomic status showed fewer days/week of physical activity, fewer sports, and lower rates of ever playing sports (50). In Chile, adults from the low-socioeconomic status are more physically inactive compared to the highest socioeconomic group (51). Additionally, individuals from lower socioeconomic backgrounds may face additional stressors associated with their social and economic circumstances, which can contribute to unhealthy coping mechanisms such as emotional eating and adopting sedentary behaviors (52–54). Consequently, these factors contribute to a self-perpetuating cycle in which lower socioeconomic status individuals are more susceptible to cardiometabolic risks, physical inactivity, reduced fitness levels, and higher rates of obesity (48, 54). Hence, this social gap in physical activity could explain differences in adolescents’ CRF and WHtR according to their school type. However, wearing SU in low-socioeconomic schools does not seem to be enough to improve CRF or WHtR.

Finally, there were no significant differences between MF and WHtR according to uniform types; nonetheless, several trends were observed. For instance, higher MF in boys from private schools and lower WHtR in boys and girls from private schools wearing SU. In this sense, strength improvement requires high specificity for its development (38, 39); nonetheless, this can be explained by more movement in their free time in adolescents who use SU, previous literature showed improved MF in school-age children with greater active commuting (55). Also, trends in WHtR may be due to the fact that physical activity is a protective factor related to obesity, and wearing SU could help to increase energy expenditure through physical activity (56).

The present study contributes to the literature showing that adolescents wearing SU present higher CRF and present some trends indicating higher MF and lower WHtR. Furthermore, enhancing these physiological measures have been related to better mental health, body composition, cognitive performance, and academic achievement (17, 18, 27, 57). Thus, a novel, low-cost strategy to boost educational performance and health parameters could be to promote SU wearing at schools. However, intervention studies are needed to support this suggestion, and particular attention must be taken to promote physical activity and physical fitness in low-socioeconomic schools and girls through this strategy.

### Strength and limitations

Some strengths of this study were the large sample of Chilean adolescents, including rural and urban areas. Also, the primary statistical analysis permitted to control of the cluster effect (i.e., school type) and to explore the association of school type and sex over our outcomes. To our knowledge, it is one of the few studies that compare the difference between uniform types with fitness and fatness indicators. These findings contribute to the published literature which shows that SU is associated with higher physical activity and, in turn, could affect relevant health indicators. Finally, although the causal relationship between higher physical activity and higher CRF due to SU must be verified, we expect the bi-directionality likelihood in our study to be low because in Chile the selection of the type of uniform by schools is independent of the student’s characteristics, and parents choose schools based on their economic circumstances.

This study has certain limitations that need to be acknowledged. One of the limitations arises from the complexity of our secondary approach, which involves analyzing three factors: sex, uniform type, and school type, along with additional covariates and differences in group sizes. Due to the intricacies involved, we were unable to meet the assumption of homoscedasticity. While there are non-parametric or robust statistical models available to address violations of homoscedasticity, not all of them align perfectly with our specific research approach. Therefore, after careful consideration, we decided to utilize ANCOVA as our chosen statistical method.

In addition, it is important to note that cardiorespiratory fitness (CRF) in this study was assessed using an indirect test rather than ergospirometry. Furthermore, the absence of variables related to nutrition and self-perception is another limitation. For future studies, it would be valuable to incorporate accelerometry to measure physical activity levels during the school day and examine its relationship with CRF, specifically by comparing the two types of school uniforms. Additionally, exploring the potential mediating effects of parental socioeconomic level, motivations and perceptions of schoolchildren, and their impact on the association between uniform type and fitness would be a relevant area for further investigation.

## Conclusion

Based on the present findings and Chilean scholarly context, this study suggests that wearing SU is linked to higher CRF compared to TU in a large sample of adolescents. Also, SU wearing does seem to be related to a favorable trend in MF and WHtR. Nonetheless, these findings are observed mainly in boys and girls from private and subsidized schools. Hence, although it is recommended that school communities encourage SU wearing due to its association with CRF, MF, and WHtR, complementary initiatives must be taken to improve results in girls and public schools. Decision-makers could use these findings as a novel strategy to improve the health of Chilean adolescents while considering the limitations of our research. It is crucial to emphasize the importance of cohesion between the education and health systems.

## Data availability statement

Publicly available datasets were analyzed in this study. This data can be found here: The dataset analyzed during this study is available on request from the Chilean government’s education quality agency.

## Ethics statement

The studies involving human participants were reviewed and approved by Education Quality Agency, Ministry of Education, Chile. Written informed consent for participation was not provided by the participants’ legal guardians/next of kin because it was a policy of the government of Chile, which was imposed on all schools in the country by the Quality of Education Agency of the Ministry of Education.

## Author contributions

CC-M: supervision the whole investigation. CC-M, RM-F, and FP: conceptualization, investigation, formal analysis, investigation, and writing–original draft. CC-M, RM-F, KS, GF, NA-F, IS, TR-A, and FR-R: review and editing and modification of original draft. All authors have read and agreed to the published version of the manuscript.

## Conflict of interest

The authors declare that the research was conducted in the absence of any commercial or financial relationships that could be construed as a potential conflict of interest.

## Publisher’s note

All claims expressed in this article are solely those of the authors and do not necessarily represent those of their affiliated organizations, or those of the publisher, the editors and the reviewers. Any product that may be evaluated in this article, or claim that may be made by its manufacturer, is not guaranteed or endorsed by the publisher.
